# Chemopotentiation of CCNU metabolites by misonidazole.

**DOI:** 10.1038/bjc.1985.110

**Published:** 1985-05

**Authors:** R. T. Mulcahy


*Br. J. Cancer* (1985), **51**, 733–735

### Short Communication

### Chemopotentiation of CCNU metabolites by misonidazole

R.T. Mulcahy

*Department of Pathology and the Cancer Center, University of Rochester, Rochester, New York 14642, USA.*

The *in vitro* cytotoxicity and *in vivo* efficacy of 1-(2-chloroethyl)-3-cyclohexyl-1-nitrosourea (CCNU) can be markedly enhanced in combination with certain radiation sensitizing compounds (reviewed by Siemann, 1982), most notably Misonidazole (MISO). CCNU, like other nitrosoureas, undergoes a spontaneous chemical decomposition to yield two active species, an alkylating chloroethyl carbonium ion as well as a carbamoylating isocyanate (Montgomery *et al.*, 1967). Alkylation is considered to be responsible for cytotoxicity (Wheeler *et al.*, 1974), while carbamoylation can account for the inhibition of protein-mediated processes such as the repair of DNA damage (Kann *et al.*, 1980) and the regeneration of intracellular glutatione (Babson & Reed, 1978). When administered *in vivo* certain nitrosoureas, including CCNU, are modified by hepatic mixed-function oxidases prior to their decomposition to therapeutically active species. In the case of CCNU, the cyclohexyl ring of the parent drug is rapidly hydroxylated at multiple sites to yield a mixture of cis- and trans- hydroxylated analogs, some of which have properties significantly different from those of the parent compound (May *et al.*, 1974; Hilton & Walter, 1975; Wheeler *et al.*, 1977). As these analogs are believed to be the immediate precursors of active moieties, it is likely that the chemopotentiation observed when MISO is combined with CCNU *in vivo* represents a composite of the interactions of MISO with the individual hydroxylated CCNUs or the damage induced by these agents. In order to determine whether MISO can potentiate the cytotoxicity of individual CCNU metabolites EMT-6/Ro tumour cells were treated *in vitro* with MISO combined with four hydroxylated metabolites of CCNU: cis: and trans-2-OH CCNU and cis- and trans-4-OH CCNU. The latter represent the major metabolites recovered from the plasma of humans and rodents treated with CCNU.

Although chemopotentiation can be induced in EMT-6/Ro cell cultures treated at oxygen tensions between fully aerobic and radiobiologically hypoxic, the magnitude of enhancement is greatest under hypoxic conditions (Mulcahy, 1984). Therefore, for these experiments EMT-6/Ro cells were treated with each of the nitrosoureas alone or in combination with 1.0 mM MISO under hypoxic conditions (<10 ppm 02). Details of the experimental methodology are described in detail elsewhere (Mulcahy *et al.*, 1984). Briefly, for each treatment group 10.0 ml of complete BME medium was gassed for 3 h with a 97% N2/3% $CO_2$ gas mixture while being stirred. At the conclusion of the gassing phase cells were injected onto each of the treatment vials. Prior to injection the cells were incubated for 10 min at a concentration of $\sim 2 \times 10^7 \mathrm{ml}^{-1}$ in a Hamilton air-tight syringe at 37°C to reduce oxygen by consumption. In separate experiments it was determined that this treatment did not influence the response of EMT-6/Ro cells to subsequent nitrosourea treatment.

To initiate exposure, various concentrations of the nitrosoureas were injected into each of the treatment vials, without interrupting gassing. The nitrosoureas were initially dissolved in absolute ethanol and were diluted 1:100 upon addition to the treatment vials. The final concentration of alcohol (1%) did not significantly influence the plating efficiency relative to controls. For those experiments in which cells were exposed to MISO and the nitrosoureas simultaneously, MISO was dissolved in complete BME at a concentration of 1.0 mM prior to the initiation of the gassing sequence. During the entire exposure period the vials were continuously purged with the gas mixture. Four hours after injection of the drugs the medium containing drugs and cells was removed from each vial. The cells were centrifuged, washed and resuspended in fresh BME. Survival was determined using a standard plating efficiency assay. Survival curves were generated by fitting the data to a linear quadratic model by least squares analysis. Dose Enhancement Factors (DEF; defined as the ratio of nitrosourea doses required to reduce cell survival to 0.001 alone or in combination with MISO) were determined from the resultant dose response curves according to the following equation:

Correspondence: R.T. Mulcahy, Box 626, 601 Elmwood Avenue Rochester, New York, 14642.
Received 15 August 1984; and in revised form 11 January 1985.

© The Macmillan Press Ltd., 1985

**Figure 1** Dose response curves for each of the monohydroxylated-CCNU metabolites (X) and CCNU (○) (a) cis-4-OH CCNU; (b) cis-2-OH CCNU; (c) trans-4-OH CCNU; (d) trans-2-OH CCNU. The dotted line in the last 3 panels represents the dose response curve for CCNU redrawn from the data graphed in the first panel (○). (+) Are survival data for EMT-6/Ro cells treated with monohydroxylated-CCNU metabolites and 1.0 mM MISO under hypoxic conditions. The data represent the pooled results of from 2–4 separate experiments for each compound.

$DEF = -\alpha_c + (\alpha_c^2 + 4\beta_c D(\alpha_m + \beta_m D))^{\frac{1}{2}}/2\beta_c D$, where $\alpha_c$, $\beta_c$ are the $\alpha$, $\beta$ parameters for cells treated with nitrosourea alone and $\alpha_m$, $\beta_m$ are the parameters for cells treated with nitrosourea and 1.0 mM MISO; D = dose.

The cytotoxicity of the trans-2-OH or trans-4-OH analogue was not significantly different from that for CCNU (analysis of covaraince; $P>0.1$). However, cis-4-OH CCNU was significantly more toxic than CCNU, while the cis-2-OH isomer was significantly less effective (analysis of covariance; $P<0.05$). Cytotoxicity of all four isomers was significantly ($P<0.01$) enhanced in combination with 1.0 mM MISO (Figure 1). DEFs (95% conf. int.) of 1.3 (1.2–1.4), 2.0 (1.8–2.2), 1.8 (1.7–1.9) and 1.7 (1.6–1.9) were produced when MISO was combined with cis-4-OH, trans-2-OH, cis-2-OH and trans-4-OH CCNU, respectively. The enhancements for the latter three drugs are comparable to those observed when MISO is combined with the parent CCNU *in vitro* (DEF = 1.6–1.8; Mulcahy *et al.*, 1984).

These studies indicate that MISO is capable of enhancing the cytotoxicity of monohydroxy-CCNU metabolites, as well as CCNU *in vitro*. Since in previous studies compounds enhanced by MISO *in vitro* have been similarly enhanced *in vivo* (Mulcahy, 1982; Mulcahy *et al.*, 1984), it seems likely that the effectiveness of the monohydroxylated analogues of CCNU, the major metabolites and immediate precursors of alkylating and carbamoylating moieties, will be potentiated in combination with MISO *in vivo*. This may be important in relation to the observations by Lee & Workman (1983) that the concentrations of the major CCNU metabolites are elevated following administration of large single doses of MISO. They maintain that the pharmacokinetic alterations observed subsequent to MISO administration are a major factor in the chemopotentiation of CCNU. While the increase in the concentration of these cytotoxic moieties undoubtedly contributes to the enhancement of CCNU cytotoxicity by large doses of MISO *in vivo*, our investigations suggest that MISO is capable of interacting with the major metabolites of CCNU in a non-pharmacokinetic fashion to enhance their cytotoxicity. We contend that this interaction may actually predominate when lower doses of MISO are employed. Since the enhancements produced when MISO is combined with hydroxylated metabolites of CCNU are, in general, comparable to those observed for CCNU itself, MISO-induced alterations in the rate of CCNU conversion or in the distribution of major monohydroxylated products are unlikely to adversely effect the enhancement of toxicity *in vivo*.

The author wishes to thank Nancy L. Martin and Gregg A. Ublacker for excellent technical assistance. Special thanks are offered to Dr V. Narayanan of the Drug Synthesis and Chemistry Branch, NCI for providing Misonidazole and Dr Thomas P. Johnston, Southern Research Institute, Alabama for the synthetic CCNU metabolites.

This work was supported by National Institutes of Health Grant CA-32374.

### References

BABSON, J.R. & REED, D.J. (1978). Inactivation of glutathione reductase by 2-chloroethyl nitrosourea-derived isocyanates. *Biochem. Biophys. Res. Comm.*, **83**, 754.

HILTON, J. & WALKER, M.D. (1975). Hydroxylation of 1-(2-chloroethyl)-3-cyclohexyl-1-nitrosourea. *Biochem. Pharmacol.*, **24**, 2153.

KANN, H.E., SCHOTT, M.A. & PETKUS, A. (1980). Effects of structure and chemical activity on the ability of nitrosoureas to inhibit DNA repair. *Cancer Res.*, **40**, 50.

LEE, F.Y.F. & WORKMAN, P. (1983). Modification of CCNU pharmacokinetics by misonidazole – A major mechanism of chemosensitization in mice. *Br. J. Cancer*, **47**, 659.

MAY, H.E., BOOSE, R. & REED, D.J. (1974). Hydroxylation of the carcinostatic 1-(2-chloroethyl)-3-cyclohexyl-1-nitrosourea (CCNU) by rat liver microsomes. *Biochem. Biophys. Res. Commun.*, **57**, 426.

MONTGOMERY, J.A., JAMES, R., MCCALBE, G.S. & JOHNSTON, T.P. (1967). The modes of decomposition of 1,3-bis(2-chloroethyl)-1-nitrosourea. *J. Med. Chem.*, **10**, 668.

MULCAHY, R.T. (1982). Chemical properties of nitrosoureas – Implications for interaction with misonidazole. *Int. J. Radiat. Oncol. Biol. Phys.*, **8**, 599.

MULCAHY, R.T., DEMBS, N.L. & UBLACKER, G.A. (1984). Enhancement of nitrosourea cytotoxicity by misonidazole *in vitro*: Correlation with carbamoylating potential. *Br. J. Cancer*, **49**, 307.

SIEMANN, D.W. (1982). Potentiation of chemotherapy by hypoxic cell radiation sensitizers – A review. *Int. J. Radiat. Oncol. Biol. Phys.*, **8**, 1029.

WHEELER, G.P., BOWDEN, B.J., GRIMSLEY, J.A. & LLOYD, H.H. (1974). Interrelationships of some chemical, physicochemical and biological properties of several 1-(2-haloethyl)-1-nitrosoureas. *Cancer Res.*, **34**, 194.

WHEELER, G.P., JOHNSTON, T.P., BOWDON, B.J., MCCALEB, G.S., HILL, D.L. & MONTGOMERY, J.A. (1977). Comparison of properties of metabolites of CCNU. *Biochem. Pharmacol.*, **26**, 2331.


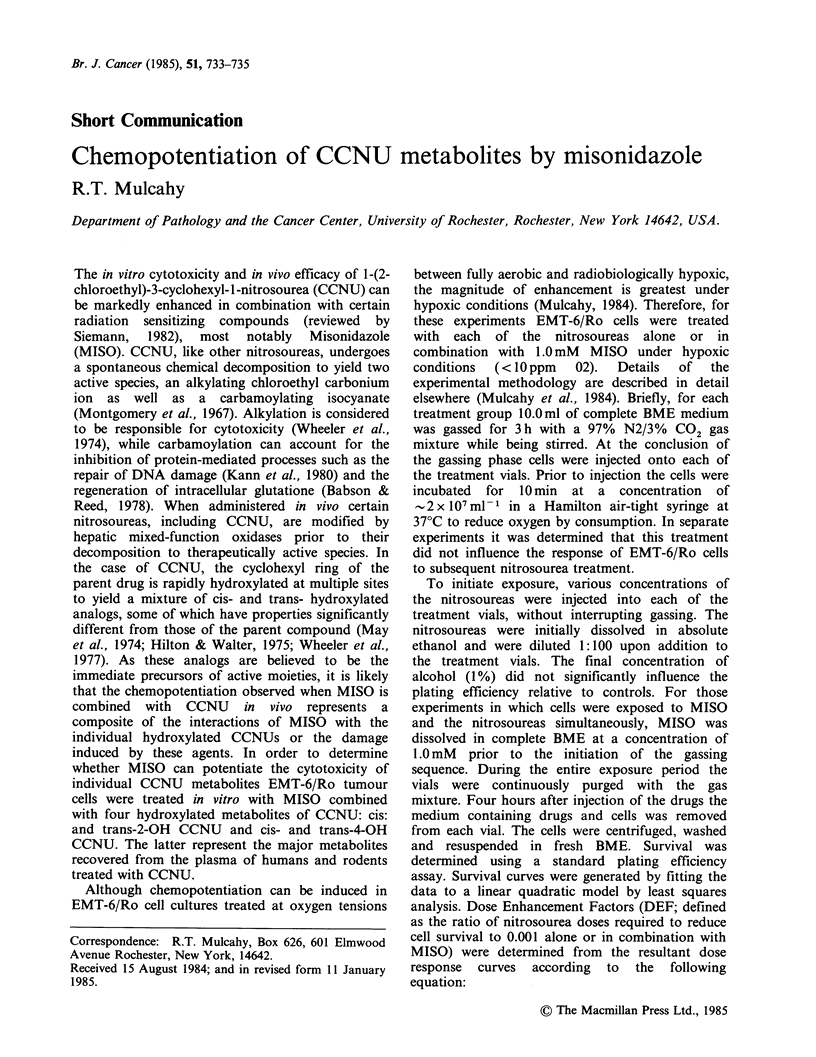

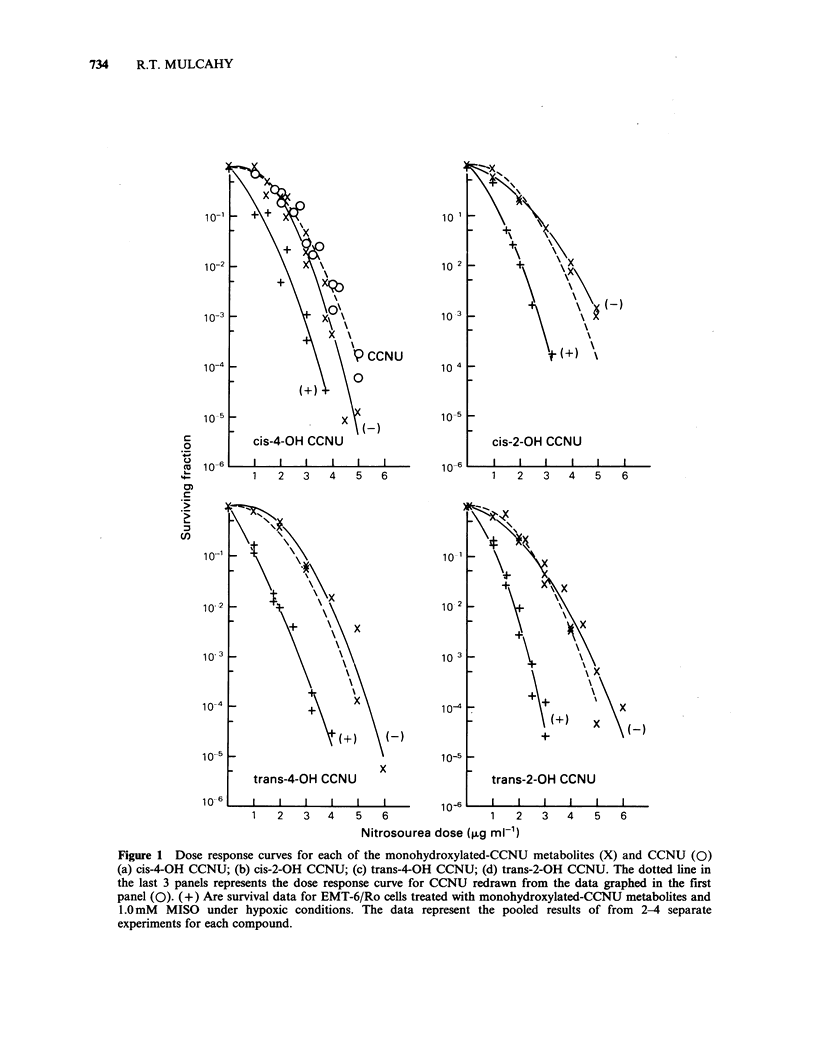

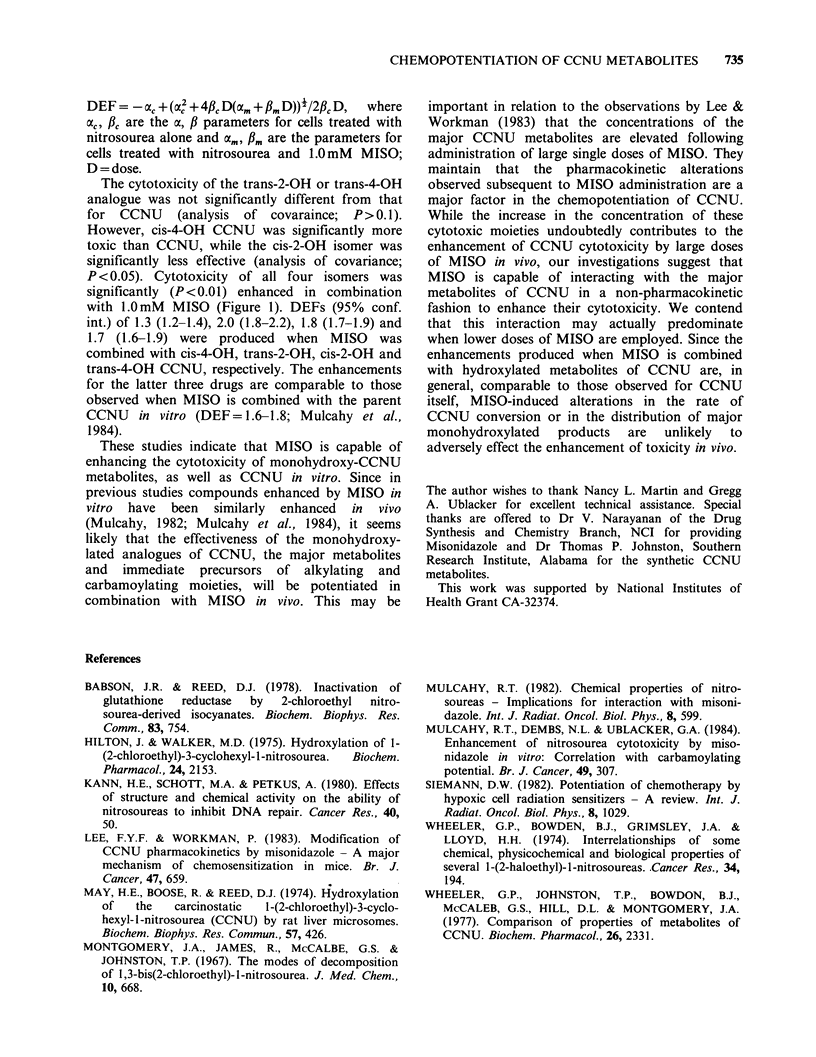


## References

[OCR_00249] Babson J. R., Reed D. J. (1978). Inactivation of glutathione reductase by 2-chloroethyl nitrosourea-derived isocyanates.. Biochem Biophys Res Commun.

[OCR_00255] Hilton J., Walker M. D. (1975). Hydroxylation of 1-(2-chloroethyl)-3-cyclohexyl-1-nitrosourea.. Biochem Pharmacol.

[OCR_00260] Kann H. E., Schott M. A., Petkas A. (1980). Effects of structure and chemical activity on the ability of nitrosoureas to inhibit DNA repair.. Cancer Res.

[OCR_00266] Lee F. Y., Workman P. (1983). Modification of CCNU pharmacokinetics by misonidazole--a major mechanism of chemosensitization in mice.. Br J Cancer.

[OCR_00272] May H. E., Boose R., Reed D. J. (1974). Hydroxylation of the carcinostatic 1-(2-chloroethyl)-3-cyclohexyl-1-nitrosourea (CCNU) by rat liver microsomes.. Biochem Biophys Res Commun.

[OCR_00278] Montgomery J. A., James R., McCaleb G. S., Johnston T. P. (1967). The modes of decomposition of 1,3-bis(2-chloroethyl)-1-nitrosourea and related compounds.. J Med Chem.

[OCR_00284] Mulcahy R. T. (1982). Chemical properties of nitrosoureas: implications for interaction with misonidazole.. Int J Radiat Oncol Biol Phys.

[OCR_00289] Mulcahy R. T., Dembs N. L., Ublacker G. A. (1984). Enhancement of nitrosourea cytotoxicity by misonidazole in vitro: correlation with carbamoylating potential.. Br J Cancer.

[OCR_00295] Siemann D. W. (1982). Potentiation of chemotherapy by hypoxic cell radiation sensitizers--a review.. Int J Radiat Oncol Biol Phys.

[OCR_00300] Wheeler G. P., Bowdon B. J., Grimsley J. A., Lloyd H. H. (1974). Interrelationships of some chemical, physicochemical, and biological activities of several 1-(2-haloethyl)-1-nitrosoureas.. Cancer Res.

[OCR_00307] Wheeler G. P., Johnston T. P., Bowdon B. J., McCaleb G. S., Hill D. L., Montgomery J. A. (1977). Comparison of the properties of metabolites of CCNU.. Biochem Pharmacol.

